# Plasmonic Imaging of Electrochemical Reactions at Individual Prussian Blue Nanoparticles

**DOI:** 10.3389/fchem.2021.718666

**Published:** 2021-09-06

**Authors:** Adaly Garcia, Kinsley Wang, Fatima Bedier, Miriam Benavides, Zijian Wan, Shaopeng Wang, Yixian Wang

**Affiliations:** ^1^ Department of Chemistry and Biochemistry, California State University, Los Angeles, Los Angeles, CA, United States; ^2^ Biodesign Center for Biosensors and Bioelectronics, Arizona State University, Tempe, AZ, United States; ^3^ School of Electrical, Computer and Energy Engineering, Arizona State University, Tempe, AZ, United States; ^4^ School of Biological and Health Systems Engineering, Arizona State University, Tempe, AZ, United States

**Keywords:** prussian blue nanoparticles, hydrogen peroxide, single entity electrochemistry, plasmonic electrochemical microscopy, surface plasmon resonance

## Abstract

Prussian blue is an iron-cyanide-based pigment steadily becoming a widely used electrochemical sensor in detecting hydrogen peroxide at low concentration levels. Prussian blue nanoparticles (PBNPs) have been extensively studied using traditional ensemble methods, which only provide averaged information. Investigating PBNPs at a single entity level is paramount for correlating the electrochemical activities to particle structures and will shed light on the major factors governing the catalyst activity of these nanoparticles. Here we report on using plasmonic electrochemical microscopy (PEM) to study the electrochemistry of PBNPs at the individual nanoparticle level. First, two types of PBNPs were synthesized; type I synthesized with double precursors method and type II synthesized with polyvinylpyrrolidone (PVP) assisted single precursor method. Second, both PBNPs types were compared on their electrochemical reduction to form Prussian white, and the effect from the different particle structures was investigated. Type I PBNPs provided better PEM sensitivity and were used to study the catalytic reduction of hydrogen peroxide. Progressively decreasing plasmonic signals with respect to increasing hydrogen peroxide concentration were observed, demonstrating the capability of sensing hydrogen peroxide at a single nanoparticle level utilizing this optical imaging technique.

## Introduction

Prussian blue, first discovered as a pigment, is composed primarily of a ferrous ion connected to a ferric ion *via* a cyanide bridge, allowing for efficient electron transfer (Kong et al., 2015; Hegner et al., 2016). The rigid and open three-dimensional lattice structure of the nanoparticle allows for the efficient cycling of alkali-ions, a key trait contributing to its reliable rechargeability (Jiang et al., 2017). Prussian blue’s characteristic blue color can be attributed to charge transfer between the two iron centers. As a result of its physical features and electronic richness (Fang et al., 2016), over the past few decades, this pigment has been used for energy storage and conversion (Jiménez-Gallegos et al., 2010; Chen et al., 2016), sensing (Karyakin et al., 1995), drug delivery (Wang et al., 2013), and catalysis (Sitnikova et al., 2014; Xuan et al., 2017; Ma et al., 2019). In particular, Prussian blue nanoparticles (PBNPs) have been demonstrated to be an effective nanocatalyst for the reduction of hydrogen peroxide (Karyakin, 1999; Karyakin et al., 2004; Mao et al., 2011; Cinti et al., 2014; Komkova et al., 2018), the overproduction and eventual accumulation of which may result in oxidative stress (Sayre et al., 2008). Sensing small amounts of hydrogen peroxide gives many insights about cells’ status and levels of oxidative stress and inflammation (Karyakin et al., 2004; Mao et al., 2011; Komkova et al., 2013).

Traditional ensemble methods provide only averaged properties of PBNPs, which limits the understanding of the properties specific to individual particles (Sardar et al., 2009; Baer et al., 2013; Trindell et al., 2019). Due to the heterogeneity in size, shape, and surface structure of nanoparticles (Seney et al., 2009; Mirkin et al., 2016; Mao et al., 2019; Trindell et al., 2019; Xie et al., 2020), analysis on a single PBNP level is thus essential to determine the relationship between the catalytic properties and the nanoparticle structure. Previous single nanoparticle analysis techniques involve the attachment of a particle to a nanoelectrode (Vakarelski and Higashitani, 2006; Holub et al., 2020), scanning probe microscopy (e.g., scanning electrochemical microscopy, scanning electrochemical cell microscopy) (Tel-vered and Bard, 2006; Kwon and Bard, 2012; Choi et al., 2020), the collision of individual nanoparticles with a microelectrode (Zhou et al., 2011) or a nanopipette (Zhou et al., 2017; Han et al., 2019), and surface/tip-enhanced Raman spectroscopy (Kurouski et al., 2015). *In situ* electron microscopy techniques, such as transmission electron microscopy (TEM) and scanning transmission electron microscopy coupled with electron energy loss spectroscopy (STEM-EELS), have also become possible recently for imaging electrochemical reactions at the nanoscale using a sealed liquid cell and provides high spatial resolution (Holtz et al., 2014; Wang et al., 2016; Zhu et al., 2020). Finally, optical techniques, such as single-molecule fluorescence microscopy (Tachikawa et al., 2011), dark field microscopy (Byers et al., 2016), electrochemiluminescence (Wilson et al., 2015), plasmonic electrochemical microscopy (Cho et al., 2015; Ngo et al., 2019; Garcia et al., 2021), and interferometric plasmonic imaging (Yang et al., 2018; Chen et al., 2020), have also been applied to study single entity electrochemistry with high temporal resolution. Specifically, plasmonic electrochemical microscopy (PEM) combines optical imaging capability with the surface sensing power of surface plasmon resonance (SPR) to enable high throughput characterization of individual nano-entities. The principle of PEM is to image with SPR the local changes (e.g., in refractive index, the thickness of deposited films, or surface charge density) associated with electrochemical reactions near an electrode surface as a function of potential and time. This technique has been well-established over the past 10 yr and applied for single nanoparticle analysis (Shan et al., 2010; Shan et al., 2012; Fang et al., 2014; Nizamov et al., 2016; Wang et al., 2017) and was recently applied to characterize the thin-layer electrochemistry at single PBNPs (Jiang et al., 2017).

**FIGURE 1:**
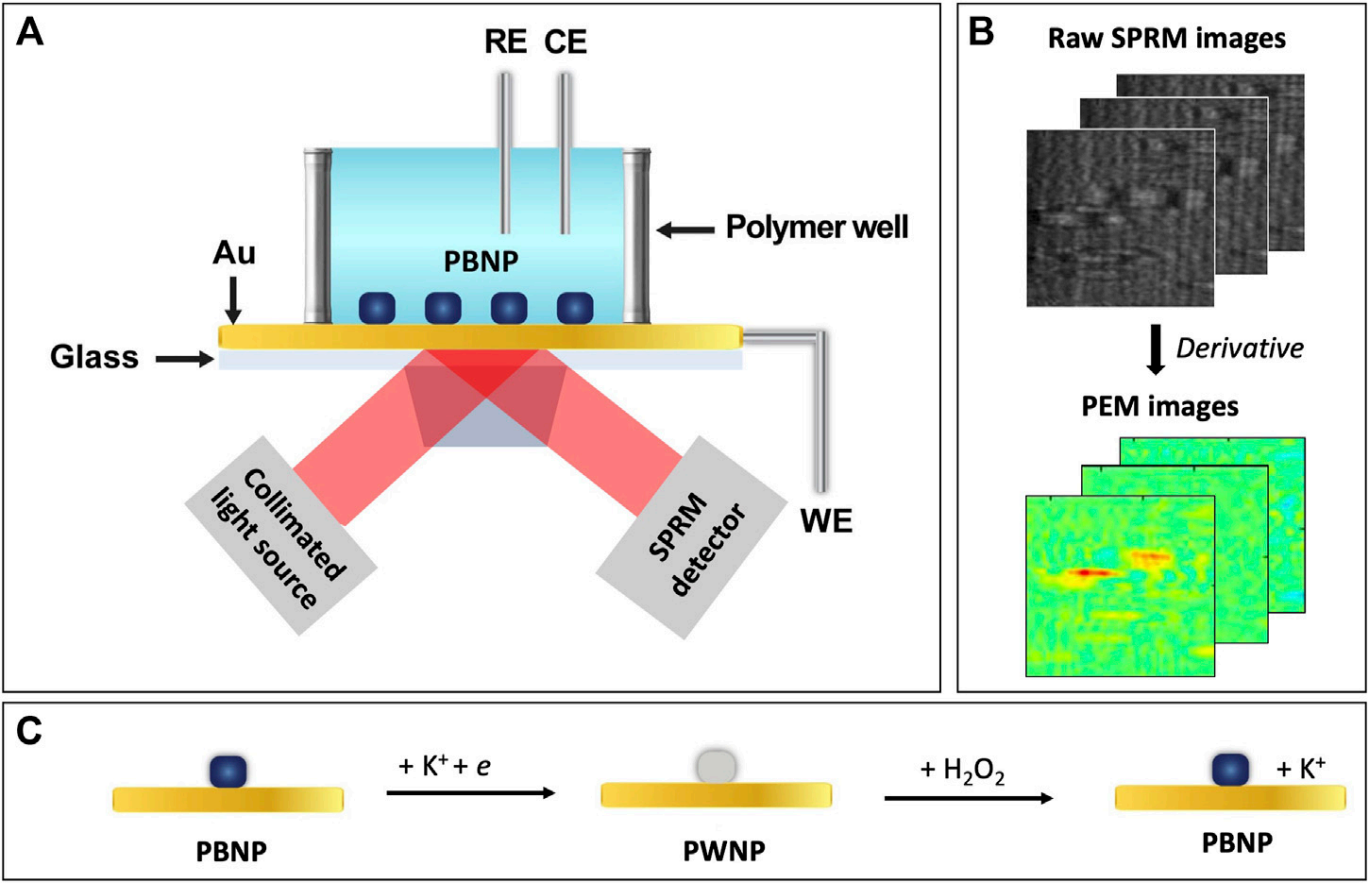
Schematic of **(A)** the PEM experimental setup, **(B)** image processing, and **(C)** the redox reaction at a PBNP.

Here we report on applying PEM to study the effect of particle structures on the electrochemistry of PBNPs and the catalytic reduction of hydrogen peroxide at individual PBNPs. As shown in Figure 1A, PBNPs were deposited on a gold sensing chip, which works as both the SPR sensing chip and the working electrode for a three-electrode electrochemical (EC) cell. The reference and counter electrodes were a chloridized Ag wire quasi-reference electrode and a Pt wire, respectively. The EC cell was mounted on a prism stage with refractive index matching oil. P-polarized light was directed onto the gold sensing chip through the prism for plasmonic excitation, and the reflected light was collected with an SPR microscopy (SPRM) detector. Electrochemical reactions on the PBNPs were controlled by directly applying a potential variation (i.e., potential sweep in cyclic voltammetry, CV) onto the gold sensing chip. Figure 1B shows representative raw SPRM images collected from a PBNPs containing sample, in which each NP generated a parabolic tail that is the summation of the partially reflected light and scattered plasmonic waves. Electrochemical reactions induce changes in the optical properties at individual PBNPs, reflected by local light intensity changes, as described by the following equation (Yu et al., 2014; Fang et al., 2016; Wang et al., 2017),

$$\frac{\text{d}I_{\text{SPR}}}{\text{d}t}=2\left|E_{\text{r}}\right|\left|E_{\text{sp}}\right|\cos\alpha \frac{\text{d}\beta }{\text{d}t}$$ (1)

Where $I_{\text{SPR}}$ is the total reflected light intensity detected in the plasmonic image, $E_{\text{r}}$ the partially reflected incident light wave, $E_{\text{sp}}$ the surface plasmonic wave, and *α* the phase difference between the two waves. *β* describes the scattering strength that depends on the nanoparticle’s optical property (e.g., refractive index), the change of which is expected to be proportional to the amount of the electrochemical reaction per unit time (reaction rate) when induced by an electrochemical reaction. Therefore, the changes in optical responses are proportional to the reaction rate and the current density, based on which, by taking the derivative of the SPRM images, PEM images can be obtained and used to extract information at individual PBNPs to plot plasmonic CVs. The use of the first order derivative of signals to study the electrochemical reactions of individual nanoparticles has been demonstrated in multiple plasmonic publications (Jiang et al., 2017; Wang et al., 2017) as well as in other optical techniques-based work such as fluorescence imaging (Guerrette et al., 2013) and dark-field scattering (Hill and Pan, 2013). Specifically, it has been demonstrated efficient for studying the electrochemistry of Prussian blue nanoparticles based on the linear correlation between the original SPRM intensity and the oxidation states of PBNPs (Jiang et al., 2017).

We investigated the redox reactions at individual PBNPs, as described by Figure 1C. Without the presence of H_2_O_2_, PBNPs can be reduced to Prussian white nanoparticles (PWNPs), which can be oxidized again to return to PBNPs. With the presence of H_2_O_2_, the reduced form, PWNPs, has a catalytic effect toward reducing H_2_O_2_ while converting back to PBNPs. Two types of PBNPs were synthesized through two different methods: type I through mixing a ferrous and a ferric salt (Qiu et al., 2007) and type II through a polyvinylpyrrolidone (PVP)-assisted facile size- and shape-controlled method (Ming et al., 2012), and their redox activities were compared and correlated to the particle structures (e.g., size and geometry). We also investigated the reduction catalytic activity of type I PBNPs with varying hydrogen peroxide concentrations and analyzed the PEM signals’ concentration dependence.

## Materials and Methods

### Chemicals and Materials

Ferric chloride, potassium ferrocyanide, potassium ferricyanide, hydrochloric acid, potassium chloride, potassium nitrate, hydrogen peroxide (3% w/v), and PVP were purchased from Fisher Scientific (Watham, MA, United States) and used without further purification. All aqueous solutions were prepared from double-deionized water (resistivity = 17.9 MΩ cm at 25°C, Barnstead Nanopure Diamond Water Purification System; APS Water, Lake Balboa, CA, United States). Silicon wells were cut from flexiPERM slides purchased from Sarstedt (Germany). The Ag/AgCl reference electrodes and Pt wire counter electrodes were purchased from Biosensing Instruments (Tempe, AZ, United States). Two types of gold sensing chips were used. The first type was bare gold sensing chips purchased from Biosensing Instruments (Tempe, AZ, United States). The second type was customized gold sensing chips fabricated with glass coverslips with grids (Bellco Glass, Inc. Vineland, NJ, United States). The coverslips are cleaned with acetone in an ultrasonic bath for 10 min, then rinsed with DI water, followed by deposition of 2 nm Cr layer and 47 nm gold layer e-beam evaporator (PVD 75, Kurt J Lesker).

### Sample Preparation

Two types of PBNPs were synthesized. Type I PBNPs (PVP free) were synthesized by mixing equimolar amounts (2 mM) of potassium ferrocyanide and ferric chloride in 0.1 mM potassium chloride and 10 mM hydrochloric acid with vigorous stirring for 12 h (Miao et al., 2007; Qiu et al., 2007) and the PBNPs were used without further treatment. Type II PBNPs were synthesized with a PVP-assisted crystallization process by mixing 3 g (or 10 g) PVP and 0.01 M potassium ferricyanide in a 0.1 M hydrochloric acid solution with vigorous stirring for 30 min (Ming et al., 2012). All type II PBNPs data was obtained from the 3 g PVP protocol unless explicitly mentioned. The solutions were then sealed in vials and placed into an oven to be heated at 80°C for 20 h. Finally, the type II Prussian blue particles were centrifuged and washed in water and ethanol, alternatively five to six times.

Both types of the PBNPs were immobilized separately on a gold sensing chip for PEM analysis no longer than a week after synthesis. Before immobilization, the gold sensing chip was cleaned by water and ethanol three times each, and a silicon well was mounted on the chip and filled with deionized water. A PBNPs stock solution (either type I or type II) was injected into the silicon well. The immobilization process was monitored with the SPRM microscope *via* the generation of characteristic parabolic tails. After observing an appropriate coverage of PBNPs, the solution was removed. The gold sensing chip was dried with nitrogen gas and further baked in an oven at 65°C for an hour to remove any residue water between the PBNPs and the gold sensing chip.

Immobilized PBNPs were characterized with atomic force microscopy (AFM) in non-contact air mode with a Park NX12 multifunctional microscopy platform equipped with a detachable AFM head (Park Systems, Seoul, South Korea). Non-contact cantilevers (PPP-NCHR, 42 N/m, 330 kHz) purchased from Park Systems were used. The instrument was operated with Smart Scan software (Park Systems, Seoul, South Korea). The acquired images were first-order flattened with XEI (Park Systems, Seoul, South Korea).

### Electrochemical Cell

A three-electrode system (a chloridized Ag wire quasi-reference electrode, Pt wire counter electrode, and the Au chip as the working electrode) was used to induce a redox reaction on the NPs. A silicon well was mounted on the gold sensing chip and filled with 1.0 M KNO_3_ solution. The exposed gold sensing chip area is approximately 0.9 cm^2^. Cyclic voltammetry was applied *via* a potentiostat (CHI610E; CH Instruments, Austin, TX, United States). In the hydrogen peroxide reduction experiments, a stock solution of 8.8 or 0.88 mM H_2_O_2_ was spiked to the electrochemical cell to reach desired concentrations. Unless otherwise stated, the voltammograms reported are for the first cycle with each sample.

### Instrumentation for Imaging

Plasmonic images were recorded with a surface plasmon resonance microscopy system (SPRm 200 Series; Biosensing Instruments, Tempe, AZ, United States) synchronized with electrochemical signals from the potentiostat (CHI610E; CH Instruments, Austin, TX, United States). The instrument was operated with ImageSPR (Biosensing Instruments, Tempe, AZ, United States). Raw SPRM image stacks were converted to *tif.* image sequences using ImageAnalysis (Biosensing Instruments, Tempe, AZ, United States). Plasmonic data were then extracted at specific locations from these image sequences using ImageJ 1.53c (Wayne Rasband, National Institutes of Health, United States) and further processed in Excel. Alternatively, the image sequences were converted to PEM images with imaging processing algorithms implemented with MATLAB (R2019b; MathWorks). Both processes (Excel and MATLAB) include taking the first order derivative and smoothing (moving average before and after taking derivative). The smoothing parameters are typically 10 and 15, before and after taking derivative, respectively, except for the type II PBNPs data, where the smoothing parameters of 15 and 60 were used, respectively. We didn’t intend to calibrate the absolute plasmonic signals in the PEM images and the plasmonic CVs to match the actual local current density. All conclusions are based on the relative intensities of the plasmonic signals within the same sample.

## Results and Discussion

### Preparation and Characterization of Prussian Blue Nanoparticles

**FIGURE 2:**
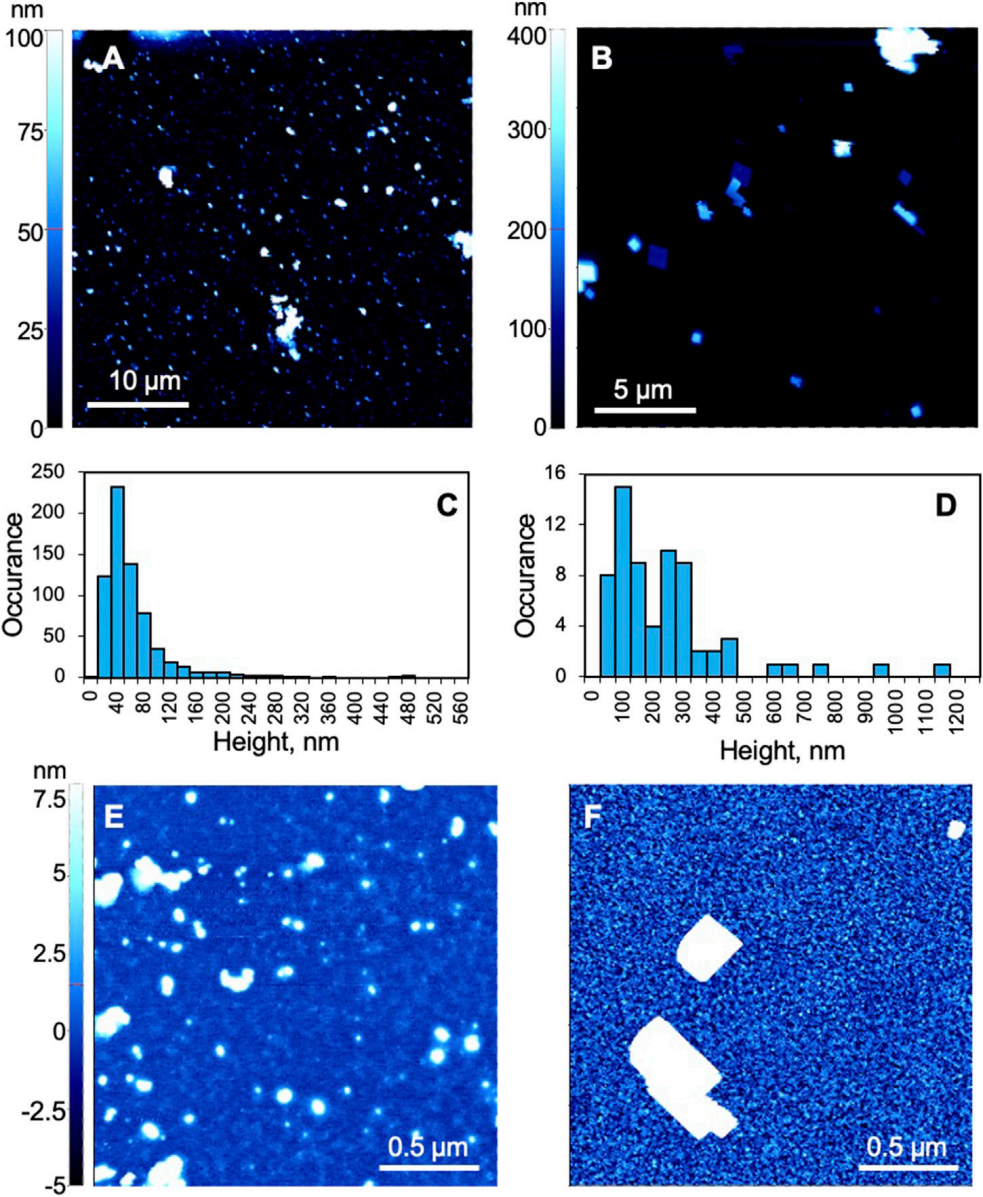
Atomic force microscopy (AFM) characterization of Prussian blue nanoparticles. **(A**,**B**,**E**,**F)** AFM images of **(A**,**E)** type I and **(B**,**F)** type II PBNPs. **(C**,**D)** Histograms of **(C)** type I and **(D)** type II PBNPs. **(C)** was based on images collected from two 40 μm × 40 μm and one 30 μm × 30 μm areas (*N* = 680), and **(D)** was based on three 20 μm × 20 μm areas (*N* = 67). All AFM figures were first-order flattened.

Two different types of PBNPs were produced following literature-reported procedures. Previous work has suggested that, when characterized with transmission electron microscopy (TEM), type I PBNPs appear as particles with a diameter around 10 nm (Miao et al., 2007) and type II from 100 to 200 nm (Ming et al., 2012). In this work, non-contact air-mode AFM was used to assess the size and geometry of the prepared PBNPs adhered to a gold sensing chip, and Figures 2A–D show that both types have wide distributions in size. The height of the majority of type I PBNPs lies between 20 and 120 nm, while that of type II lies between 50 and 500 nm, indicating type II are generally larger than type I in size. When comparing the particle geometry, type II PBNPs are more shape-controlled (i.e., cubical structure) than type I PBNPs. However, when focusing on the background of the chips, the roughness is significantly larger in the type II sample compared to the type I sample, as shown in Figures 2E,F. Excluding the PBNPs areas, Figure 2E (type I) has a peak-to-valley average of 3.7 nm, and Figure 2F (type II) 11.9 nm. The significantly larger background roughness present in type II samples was attributed to residues from PVP-assisted synthesis. The effect on electrochemical activities of these PBNPs will be discussed in the following section.

### Comparison of Electrochemistry Between Two Types of PBNPs

**FIGURE 3:**
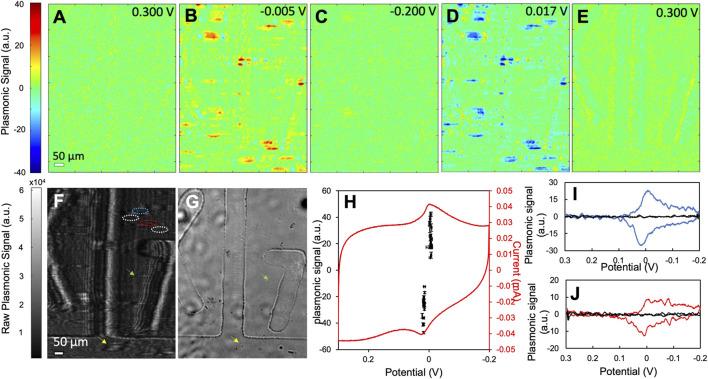
The electrochemical activity of type I PBNPs. **(A**–**E)** Representative PEM images of type I PBNPs at different potentials. **(F)** The raw SPRM image of type I PBNPs coated on a gold sensing chip with grids and **(G)** the bright field image of the same area as **(F)**. **(H)** The potentiostat CV of the entire electrode surface and **(I**,**J)** plasmonic CVs from two PBNPs (blue and red solid lines) as labeled in **(F)** (blue and red dashed circled areas) and their neighboring background areas (black solid line, labeled as white dashed circled area in **F)**. The black data points in **(H)** represent the oxidation and reduction peaks’ potentials and intensities from plasmonic CVs of 16 different PBNPs. These data points are presented with mean and standard deviation measured from four different cycles. Scanning rate: 0.05 V/s. Solution: 1.0 M KNO_3_.

**FIGURE 4:**
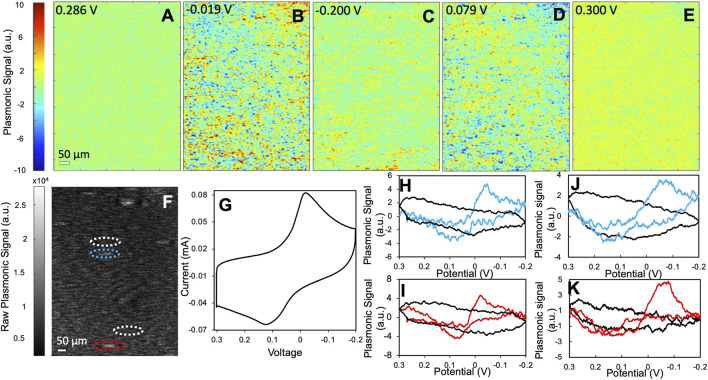
The electrochemical activity of type II PBNPs. **(A**–**E)** Representative PEM images of type II PBNPs at different potentials. **(F)** Raw SPRM image of type II PBNPs. **(G)** Potentiostat CV of the entire electrode and **(H**,**I)** plasmonic CVs from two PBNPs (blue and red) and their neighboring background areas (black) as labeled in **(F)**, and **(J**,**K)** plasmonic CVs from two type II PBNPs (blue and red) synthesized from 10 g PVP protocol and their neighboring background areas (black). Scan rate: 0.05 V/s. Solution: 1.0 M KNO_3_.

Using PEM, we obtained the CVs of the two types of PBNPs at the individual nanoparticle level and compared their activities in Figure 3 (type I) and Figure 4 (type II). Figures 3A–E show several snapshots of the PEM images of a group of type I PBNPs at different potentials, where the image contrast represents the derivative of the original SPRM images. The PEM video of the entire reduction and oxidation processes is attached in the supporting information (SI) listed as Supplementary Video S1. At 0.300 V, where the CV scan begins, PEM image contrast is minimal because the potential is very far away from the redox potential of Prussian blue, and no electrochemical reaction occurs (Figure 3A). As the potential decreases toward the reduction potential, contrast (red) in the PEM images begins to develop as the reduction of PBNPs to PWNPs occurs (Supplementary Video S1). The positive contrast reaches a maximum at −0.005 V (Figure 3B). As the potential continues to decrease, the PEM contrast decreases and finally disappears at −0.200 V (Figure 3C), corresponding to the completion of reduction of PBNPs. When the potential cycles back, the contrast is inverted (blue), which reflects the oxidation of PWNPs back to PBNPs. The maximum negative contrast occurs at about 0.017 V (Figure 3D) and disappears again when the potential cycles back to 0.300 V (Figure 3E). The potentials at which the maximum contrasts are observed correspond to the peak potentials in the potentiostat CV (Figure 3H) that represents the electrochemical activity of the entire electrode surface. The closeness of the reduction and oxidation peak potentials and the symmetrical shape of the CV is indicative of the thin-film electrochemistry at PBNPs, as reported previously (Jiang et al., 2017).

The plasmonic CVs of individual PBNPs were obtained by extracting signals at the tail area of each particle. Please note that a gold sensing chip with grids (in the form of boxes with letters and numbers) was used in this test for aligning the raw SPRM image (Figure 3H) with the bright field image (Figure 3G). Utilizing a chip with grids is to confirm the existence of particles at the position of the tails in the SPRM raw images, as demonstrated by the two pairs of arrows pointing to the position of two PB particles. This design also makes it possible in the future to correlate the PEM image with additional nanoparticle characterization with AFM, which is currently being investigated. Two PBNPs were selected as examples and outlined in the raw SPRM image (Figure 3F). Figure 3I shows the plasmonic CVs from the nanoparticle (blue) outlined with a blue dashed circle and the neighboring background area with no presence of PBNPs (black) outlined with a white dashed circle. The latter shows no plasmonic signal, which confirms that the reduction and oxidation peaks are due to PBNPs. This observation was further confirmed by the plasmonic CVs from the second PBNP and its adjacent background area in Figure 3J. Compared to the potentiostat CV, single PBNP CVs reveal sharper reduction and oxidation peaks and are less affected by the large charging current observed in the potentiostat CV. This is likely due to the higher sensitivity of SPR to local refractive index change caused by the color change from blue to colorless compared to local surface charge density change and demonstrated that SPR/PEM is selectively sensitive to the electrochemical conversion between PBNPs and PWNP, and therefore an ideal tool for investigating this system. The peak potentials and intensities of 16 different particles measured from four cycles are plotted in Figure 3H. The variation in peak potentials is relatively minor. All potential values overlap well with the peaks in the potentiostat CV, which further demonstrated the validity of this imaging technique for studying the PB/PW reaction. The wide distribution of peak intensities emphasizes particle heterogeneity. We also noticed that the density of particle signals in PEM is much less than that reflected by the AFM image in Figure 2A, which indicated that with the current setting, only relatively large PBNPs (>100 nm in height) could be detected in PEM. The detection limit can be improved in the future by averaging over multiple cycles that can enhance the signal-to-noise ratio and enable the detection of smaller particles.

To further investigate the effect of particle structures, we applied the same protocol to type II PBNPs that are larger and more regularly shaped. Figures 4A–E show several snapshots of the PEM images of a group of type II PBNPs at different potentials. The PEM video of the entire reduction and oxidation processes is attached in the SI listed as Supplementary Video S2. The PEM image contrast is initially minimal (Figure 4A) and becomes more obvious between 0 and −0.05 V (Figure 4B). As the potential continues to decrease, the PEM contrast decreases and finally disappears at −0.200 V (Figure 4C). When the potential cycles back, the negative contrast appears between 0.05 and 0.10 V (Figure 4D) and disappears again when the potential cycles back to 0.300 V (Figure 4E, Supplementary Video S2). Due to the poor signal-to-noise ratio, much higher smoothing parameters (15 and 60 before and after derivatives, respectively) were applied. Although type II PBNPs were significantly larger than type I (Figure 2), their PEM signal was much weaker and noisy, with significantly less image contrast at individual nanoparticles. As a result, it was difficult to identify the exact potentials at maximum contrast. One possible cause is the presence of PVP residues which block the surface of PBNPs and hinders the penetration of potassium ions into the Prussian blue lattice. Moreover, the PVP residue between the PBNPs and the gold sensing chip affects the electric contact and consequently the induction of electrochemical reactions at these PBNPs. Another possible cause would be the regular cubic geometry of type II PBNPs compared to the more porous and irregular structures of type I that provide a larger contact area. The hindering effect from the PVP residue and the particle structure was also reflected by the increased separation between reduction and oxidation peaks (∼0.1 V) in the potentiostat CV (Figure 4G). The plasmonic CVs from two individual PBNPs and their neighboring background areas are displayed in Figures 4H,I. The peak separation (0.15–0.2 V) is even larger than that observed from the potentiostat CV. Again, this feature can be attributed to the increased contact resistance between the gold sensing chip and the nanoparticles and the PVP residues on the particle surface. A pair of plasmonic CVs from a different type II sample obtained with 10 g PVP (Figures 4J,K) show even weaker signals and broader peak separation, which further demonstrated the interference from PVP. Similar to the type I PBNPs, the type II PBNPs also showed heterogeneity in peak intensities. Moreover, the shape of the single PBNP plasmonic CVs can potentially provide information about the surface structure of the PBNPs, while the exact correlation remains an open question and requires further studies.

### Sensing of H_2_O_2_ at Individual PB Particles

**FIGURE 5:**
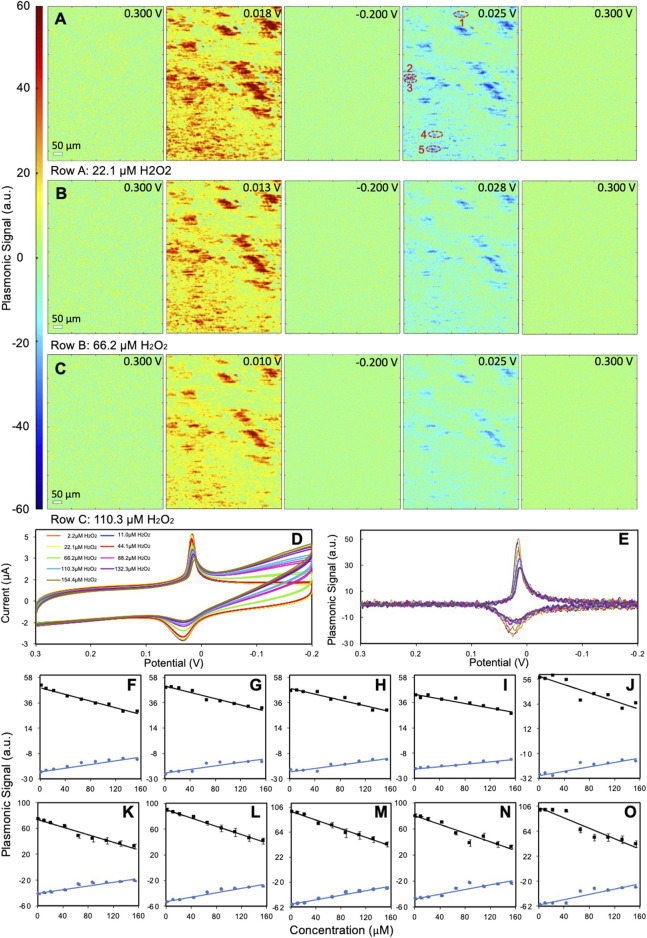
Sensing of H_2_O_2_ at type I PBNPs. **(A**–**C)** Representative PEM images of type I PBNPs with the presence of **(A)** 22.1 µM H_2_O_2_, **(B)** 66.2 µM H_2_O_2_, and **(C)** 110.3 µM H_2_O_2_ at different potentials. **(D)** Potentiostat CV of the entire electrode and **(E)** plasmonic CVs from PBNP-1 labeled in **(A)** for different H_2_O_2_ concentrations as shown in the legend. **(F**–**O)** Relationship between the plasmonic signals at redox peaks and the H_2_O_2_ concentrations at five different PBNPs (1–5 labeled in **A)** from **(F**–**J)** one cycle at the scan rate of 0.02 V/s and four cycles at the scan rate of 0.05 V/s **(K**–**O)**, respectively. Solution: 1.0 M KNO_3_ + *x* µM H_2_O_2_.

Due to the poor PEM sensitivity of type II PBNPs, we chose type I PBNPs for hydrogen peroxide reduction analysis. We monitored the electrochemical activities of PBNPs in the presence of hydrogen peroxide at nine different concentrations to explore the PEM signal’s concentration dependence at individual type I PBNPs. Figures 5A–C show several snapshots of the PEM images of a group of type I PBNPs at different potentials with 22.1 µM H_2_O_2_, 66.2 µM H_2_O_2_, and 110.3 µM H_2_O_2_ as examples. The PEM videos of the entire reduction and oxidation processes for all three trials are attached in the SI listed as Supplementary Videos S3–S5. The second and fourth columns of all three trials list the PEM images with the highest positive and negative contrast, respectively, from which a general decrease in contrast can be observed with increasing H_2_O_2_ concentrations. This is also consistent with the potentiostat and plasmonic CVs. Figure 5D shows the potentiostat CVs of the entire electrode for all nine H_2_O_2_ concentrations. Both the reduction and oxidation peaks associated with PBNP-PWNP conversion decrease when increasing the amount of H_2_O_2_. A similar phenomenon was observed from the plasmonic CVs extracted from PBNP-1 (Figure 5E). The reduction and oxidation peaks in the plasmonic CV show clearly the gradual decrease in plasmonic signal at the peak potentials, especially at the reduction peaks. This is due to the catalytic effect toward reducing H_2_O_2_ from PWNPs that convert PWNPs back to PBNPs, which consequently decreases the net change from PBNPs to PWNPs sensed by PEM (Mao et al., 2011). Meanwhile, a new reduction current starts to appear around −0.2 V in the potentiostat CV (Figure 5D), and its intensity increases with the increasing H_2_O_2_ concentration, which has been traditionally used for quantitative sensing of H_2_O_2_. However, this reduction peak is not observable in the plasmonic CVs (Figure 5E), likely due to the reduction of H_2_O_2_ not involving an observable refractive index change. There is also a noticeable continuous negative shift in the reduction peak potential with the addition of H_2_O_2_. This shift correlates with the shift in the potential that maximum peak contrast was observed in the representative PEM images (Figures 5A–C). The minor shift in potential is attributed to the usage of a quasi-reference silver electrode.

Given that the H_2_O_2_ reduction current in the potentiostat CVs is not observable in single PBNP plasmonic CVs, we investigated the concentration dependence of the plasmonic signals at the PBNP-PWNP conversion peaks. The maximum plasmonic intensities from the plasmonic CVs of five different PBNPs (as labeled in Figure 5A) were plotted versus the concentration of H_2_O_2_ added for both the reduction and oxidation peaks (Figures 5F–J). Despite the difference in the slope and sensing capacity, most PBNPs have shown a good linear concentration dependence to H_2_O_2_ (*R* ^2^ = 0.92 ± 0.06 for reduction peaks; *R* ^2^ = 0.90 ± 0.05 for oxidation peaks). We also plotted the values measured from the same PBNPs at a higher scan rate (0.05 V/s) with four cycles in Figures 5K–Q to further explore the sensing capacity, which shows a slight improvement in the linearity (*R* ^2^ = 0.95 ± 0.04 for reduction peaks; *R* ^2^ = 0.91 ± 0.05 for oxidation peaks). Overall, the values measured from reduction peaks are preferred to obtain more reliable data. Another important consideration is the linear range of detection. Because the PEM signal responds to H_2_O_2_ in a negative feedback mode, there is an upper detection limit that can be estimated based on the calibration equations from all listed particles. For plasmonic CVs obtained at 0.02 V/s, the upper limits are 369 ± 39 μM and 340 ± 42 μM, based on reduction and oxidation peaks, respectively, while for CVs at 0.05 V/s, the upper limit is 255 ± 16 μM and 286 ± 17 μM. We expect the upper detection limit to be associated with the particle structure (e.g., size, geometry, surface structure) and electrochemical measurement conditions (e.g., scan rate), which requires further studies. The lower detection limit can be estimated based on the noise level affected by the data recording and analysis process. In the current experimental setting with smoothing parameters of 10 and 15 before and after taking derivatives, the peak-to-peak noise is around 4 a.u., which leads to the lower detection limit of 30 ± 6 μM (reduction) and 55 ± 11 μM (oxidation) for 0.02 V/s, and 12 ± 2 μM (reduction) and 22 ± 3 μM (oxidation) for 0.05 V/s. The detection limit could be improved by further smoothing the data or averaging plasmonic CVs from multiple scans.

## Conclusion

By synthesizing two types of Prussian blue nanoparticles, type I and type II, we examined and compared their structures and electrochemical activities using AFM and PEM. Type I PBNPs exhibited better PEM sensitivity due to their smaller size, less regular structure, and PVP-free surface. We imaged the reduction of hydrogen peroxide at varying concentrations at type I Prussian blue nanoparticles and observed a linear relationship between the plasmonic peak signals and the corresponding concentrations of H_2_O_2._ This work incorporated plasmonic imaging and electrochemical sensing successfully to demonstrate the capability of investigating electrochemical reactions at Prussian blue nanoparticles and their sensing capability to H_2_O_2_ at the single nanoparticle level. We also showed that sensing capacity is highly dependent on individual nanoparticles of selection, the electrochemical measurement conditions, and the image collection and analysis. To fully elucidate the interrelation between an individual nanoparticle’s structures (crystal facets and microstructures) and its catalytic capabilities, future studies may benefit from combining high-resolution structural imaging tools such as atomic force microscopy, transmission electron microscopy, or scanning transmission electron microscopy to obtain atomic-level information from the same nanoparticles imaged with PEM.

## Data Availability

The original contributions presented in the study are included in the article/Supplementary Material, further inquiries can be directed to the corresponding authors.
